# Cost-effectiveness of first-line pembrolizumab monotherapy in PD-L1–high metastatic non-small cell lung cancer in Australia: evidence from trials and real-world practice

**DOI:** 10.1007/s11096-026-02202-3

**Published:** 2026-07-27

**Authors:** Srinivas Teppala, Juhee Koo, Edwin C. K. Tan, Stephen Clarke, Christine Y. Lu

**Affiliations:** 1 School of Pharmacy, Faculty of Medicine and Health, The University of Sydney, Camperdown, NSW Australia; 2 Kolling Institute, The University of Sydney and the Northern Sydney Local Health District, St Leonards, NSW Australia; 3 Charles Perkins Centre, The University of Sydney, Camperdown, NSW Australia; 4 Department of Medical Oncology, Royal North Shore Hospital, St Leonards, NSW Australia; 5 Northern Clinical School, The University of Sydney, St Leonards, NSW Australia

**Keywords:** Australia, Cost-effectiveness analysis, Non-small cell lung carcinoma, Real-world evidence, Pembrolizumab

## Abstract

**Introduction:**

Pembrolizumab monotherapy improves survival in advanced non-small cell lung cancer (NSCLC) with programmed death ligand 1 (PD-L1) tumour proportion score (TPS) ≥ 50%. However, survival in randomised trials may not fully reflect outcomes in routine practice, with implications for economic evaluation.

**Aim:**

To evaluate the cost-effectiveness of pembrolizumab monotherapy compared with platinum-based chemotherapy from an Australian health-payer perspective, integrating both randomised controlled trial (RCT) and real-world evidence (RWE).

**Method:**

A semi-Markov model with three health states (progression-free, progressed disease, death) was developed over a 3-year horizon. Clinical inputs were derived from KEYNOTE-024 for the RCT scenario and from nationwide Australian real-world data for the RWE scenario, with chemotherapy outcomes retained from the pivotal trials in both scenarios. Direct medical costs (2024 AU$) and quality-adjusted life years (QALYs) were estimated. Incremental cost-effectiveness ratios (ICERs) were calculated, probabilistic and deterministic sensitivity analyses conducted.

**Results:**

Compared with chemotherapy, pembrolizumab monotherapy was associated with incremental costs of AU$87,399 (RCT-based) and AU$75,935 (RWE-based), and incremental QALY gains of 0.23 and 0.11, respectively. Corresponding ICERs were AU$385,561/QALY (RCT) and AU$705,729/QALY (RWE). At a AU$75,000/QALY willingness-to-pay (WTP) threshold, the probability of cost-effectiveness was < 1% in both scenarios. Differences in overall survival between trial and real-world cohorts likely contributed to the variation in ICERs.

**Conclusion:**

At current pricing and Australian WTP thresholds, pembrolizumab monotherapy is unlikely to be cost-effective, with RWE-informed scenarios yielding less favourable cost-effectiveness estimates than from trial data. These findings highlight that cost-effectiveness estimates may be sensitive to differences between trial-based and real-world effectiveness inputs.

**Supplementary Information:**

The online version contains supplementary material available at https://doi.org/10.1007/s11096-026-02202-3.

## Impact statements

- Real-world evidence provides complementary insights into clinical effectiveness in routine practice compared with randomised clinical trial data. Using both pivotal trial data (KEYNOTE-024) and Australian nationwide real-world evidence, this study evaluates the cost-effectiveness of pembrolizumab monotherapy in metastatic NSCLC with high PD-L1 expression.
- We found that differences in overall survival between trial and routine practice populations can materially alter economic conclusions.
- Pembrolizumab monotherapy is unlikely to be cost-effective in the treatment of mNSCLC with high PD-L1 expression. These findings highlight the importance of integrating real-world data alongside trial results when assessing the economic value of immune checkpoint inhibitors.

## Introduction

Lung cancer is the most frequently diagnosed cancer and the leading cause of cancer-related mortality worldwide [1]. In Australia, approximately 13,563 new cases and 8,540 deaths occur annually [2]. Non-small cell lung cancer (NSCLC) accounts for roughly 85% of all lung cancers [3], with most patients presenting with advanced or metastatic disease at diagnosis, resulting in a 5-year overall survival (OS) rate as low as 3.2% [4].

Traditionally, platinum-based chemotherapy, most commonly carboplatin or cisplatin combined with agents such as pemetrexed, represented the standard first-line treatment for advanced NSCLC [5]. However, survival outcomes with chemotherapy alone are modest, with median OS typically less than one year [6]. The advent of immune checkpoint inhibitors has fundamentally reshaped the treatment landscape. By targeting inhibitory immune pathways such as programmed cell death 1/ programmed cell death ligand 1 (PD-1/PD-L1) and cytotoxic T-lymphocyte antigen 4 (CTLA-4), ICIs restore anti-tumour activity and have demonstrated substantial survival benefits compared with chemotherapy [7].

In patients with advanced non-squamous NSCLC without targetable driver mutations and PD-L1 tumour proportion score (TPS) ≥ 50%, pembrolizumab monotherapy is now recommended as the preferred first-line therapy in international clinical guidelines [5, 8, 9]. These recommendations are largely informed by the landmark KEYNOTE-024 trial, which demonstrated significantly improved OS compared with platinum-based chemotherapy (median 26.3 vs. 13.4 months) [10].

Despite their methodological rigor, randomised controlled trials (RCTs) often enrol highly selected patient populations and have limited follow-up durations, which may reduce generalisability to routine clinical practice [11]. For example, KEYNOTE-024 excluded patients with poor performance status or significant comorbidities, including active autoimmune diseases [12]. Emerging real-world evidence (RWE) suggests that OS among patients receiving pembrolizumab in routine practice may be shorter than in trials (median 13.2 months vs. 26.3 months) [13]. These discrepancies emphasise the importance of incorporating RWE to better characterise treatment effectiveness in broader, clinically representative populations.

The economics of pembrolizumab monotherapy in high PD-L1 mNSCLC is uncertain. Evaluations from U.S. [14, 15], Switzerland [16, 17], France [18] and Hong Kong [19] suggest cost-effectiveness, while those from UK [20], China [21] and Singapore [22] indicate that pembrolizumab offers poor value for money. These previous studies were based on trial-based inputs. The distinction between trial efficacy and real-world effectiveness, however, has important implications for health economic evaluations. Cost-effectiveness analyses underpinning reimbursement decisions frequently rely on RCT data alone, as exemplified by funding recommendations for pembrolizumab monotherapy by Australia’s Pharmaceutical Benefits Scheme (PBS) [23] and the UK’s National Institute for Health and Care Excellence (NICE) [24]. However, prior studies in other therapeutic areas have demonstrated that incorporating real-world data can meaningfully alter cost-effectiveness estimates [25, 26]. Given the high cost of immunotherapy, pembrolizumab exceeds AU$7,000 per dose in Australia [27], and the substantial economic burden of lung cancer, with management costs estimated at approximately AU$448 million annually [28], robust real-world economic evaluations are essential to ensure that funding decisions accurately reflect clinical effectiveness and support efficient allocation of healthcare resources.

### Aim

This study aimed to evaluate the cost-effectiveness of first-line pembrolizumab monotherapy versus platinum-based chemotherapy in advanced non-squamous NSCLC with PD-L1 TPS ≥ 50%, incorporating both RCT data and RWE from an Australian health system perspective.

## Method

### Model structure

**Fig. 1:**
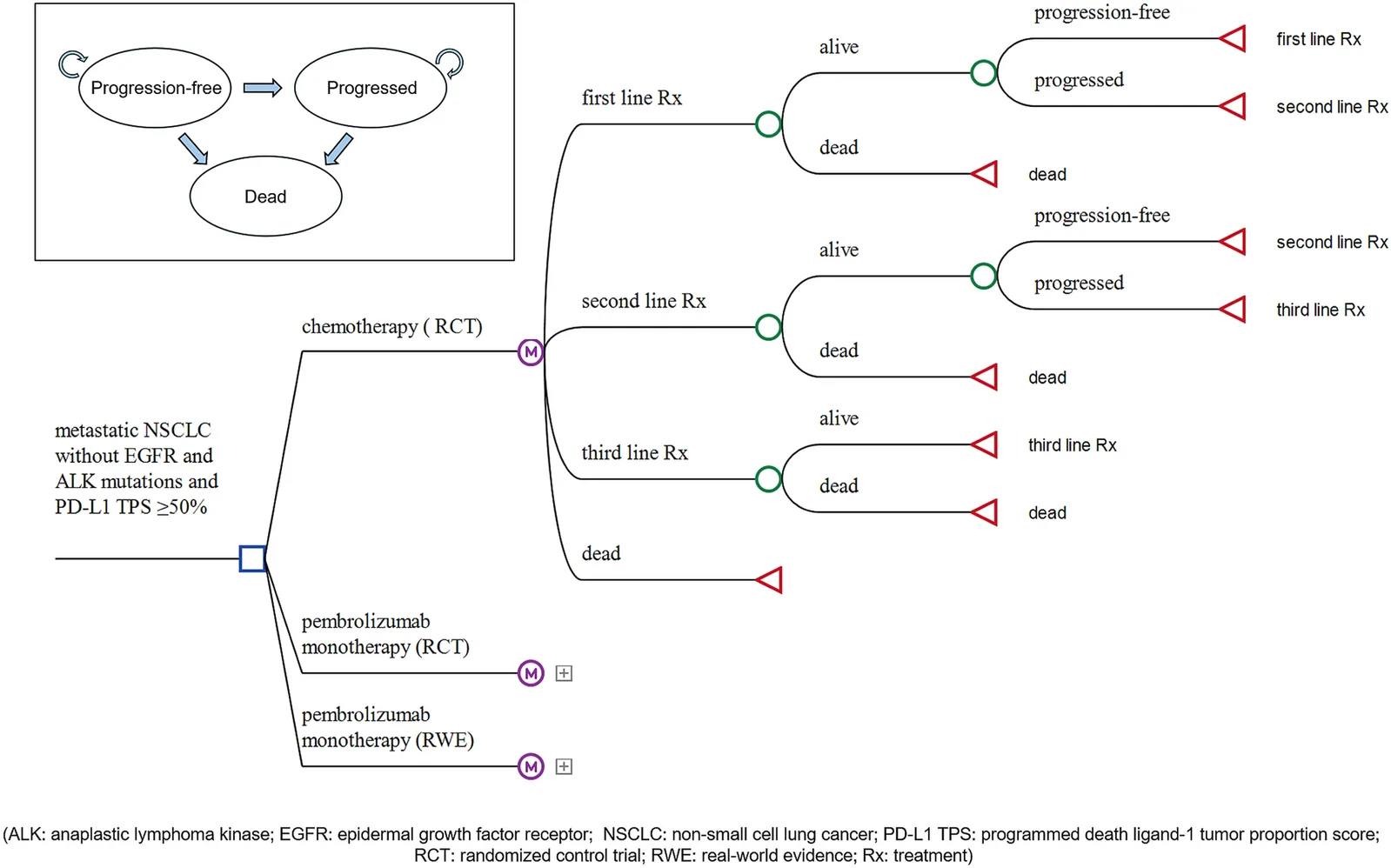
Decision tree of the cost-effectiveness model

A cost-utility analysis was conducted to compare first-line pembrolizumab monotherapy with platinum-based chemotherapy in patients with metastatic non-oncogene addicted NSCLC and PD-L1 TPS ≥ 50% from an Australian health payer perspective. A semi-Markov state-transition model was developed incorporating a decision tree followed by three mutually exclusive health states: progression-free disease, progressed disease, and death (Fig. 1). Monthly cycles were applied. Three treatment strategies were examined: (1) platinum-based chemotherapy (standard of care; SoC); (2) pembrolizumab monotherapy informed by progression-free survival (PFS) and OS data from KEYNOTE-024 [10]; and (3) pembrolizumab monotherapy informed by real-world median treatment duration (mTTD; used as a proxy for duration of first-line treatment benefit in the absence of directly observed progression data [29]) and OS estimates derived from an Australian population-based study [13]. This scenario was included to explore the impact of alternative effectiveness estimates for pembrolizumab under routine clinical practice conditions and is intended as a scenario analysis rather than a full real-world comparative effectiveness evaluation. mTTD and OS were treated as independent observed outcomes and were not mathematically linked in the estimation of health-state transitions or post-progression survival. These real-world estimates reflect outcomes in a treated mNSCLC population in which PD-L1 TPS was not available at the individual level. In Australia, pembrolizumab monotherapy is predominantly prescribed for PD-L1 TPS ≥ 50% disease under PBS reimbursement criteria, suggesting that the real-world cohort is likely enriched for PD-L1-high patients despite the absence of individual-level biomarker data. Chemotherapy outcomes in the RWE scenario were retained from KEYNOTE-024 due to the absence of a contemporaneous real-world chemotherapy comparator.

Patients assigned to the chemotherapy strategy were assumed to receive a platinum-based doublet (carboplatin or cisplatin combined with pemetrexed, gemcitabine, or paclitaxel) for up to four cycles, reflecting regimens used in routine clinical practice and permitted in KEYNOTE-024 [10, 30]. Patients experiencing disease progression were assumed to receive second-line docetaxel plus nivolumab for up to six cycles [31], followed by basic supportive care after subsequent progression [8, 9]. In the two pembrolizumab monotherapy strategies (RCT-based and RWE-based), first-line chemotherapy was replaced with pembrolizumab 200 mg administered intravenously every three weeks for up to 35 cycles. Subsequent treatment pathways mirrored those of the chemotherapy arm. Treatment sequencing and duration were consistent with KEYNOTE-024 protocols [12] and aligned with European Society of Medical Oncology (ESMO) clinical practice recommendations for advanced non-oncogene addicted NSCLC [8, 9]. Concomitant medications, including folic acid, vitamin B12, and corticosteroids (e.g., dexamethasone), were included where applicable. Patients transitioned among three mutually exclusive health states: progression-free, progressed disease, and death. Transitions were governed by health-state-specific survival estimates derived from the respective RCT or real-world data sources [10, 13].

### Model inputs

Model parameters are summarised in Table 1. Baseline patient age in the base-case analysis was derived from the KEYNOTE-024 trial population and rounded to 65 years [10]. For first-line therapy, median PFS was 5.5 months for standard chemotherapy and 7.7 months for pembrolizumab using RCT-based inputs [10], and 11.4 months for pembrolizumab using RWE [13]. Corresponding median OS values were 13.4 months for chemotherapy [10], 26.3 months for RCT-based pembrolizumab [10], and 13.2 months for RWE-based pembrolizumab [13]. Chemotherapy outcomes were sourced from KEYNOTE-024. Patients progressing to second-line therapy were assumed to receive docetaxel plus nivolumab, with a median PFS of 4.0 months [31]. Patients receiving basic supportive care were assumed to remain in that health state or transition to death, with a maximum duration of six months [32]. Death was modelled as an absorbing state. Median survival estimates were converted to monthly transition probabilities for use in the semi-Markov model, assuming constant hazards within cycle intervals.

**Table 1:** Summary of model input parameters

Parameter	Base value	Range/SD	Distribution	Sources
Age of the patient, years	65	34–84	Normal	Reck et al. [10]
PFS with first line chemotherapy, months	5.5	–	Exponential	Reck et al. [10]
PFS with first line pembrolizumab monotherapy: RCT, months	7.7	–	Exponential	Reck et al. [10]
mTTD with first line pembrolizumab monotherapy: RWE, months	11.4	–	Exponential	Yiu et al. [13]
PFS with second line docetaxel plus nivolumab, months	4.0	–	Exponential	Wang et al. [31]
OS with first line chemotherapy, months	13.4	–	Exponential	Reck et al. [10]
OS with first line pembrolizumab monotherapy: RCT, months	26.3	–	Exponential	Reck et al. [10]
OS with first line pembrolizumab monotherapy: RWE, months	13.2	–	Exponential	Yiu et al. [13]
Hazard ratio for disease progression in patients aged < 65 years: RCT	0.61	0.40–0.92	Gamma	Reck et al. [12]
mTTD in patients aged < 65 years: RWE, months	11.2	–	Exponential	Yiu et al. [13]
Hazard ratio for disease progression in patients aged ≥ 65 years: RCT	0.45	0.29–0.70	Gamma	Reck et al. [12]
mTTD in patients aged ≥ 65 years: RWE, months	11.5	–	Exponential	Yiu et al. [13]
Hazard ratio for disease progression in men: RCT	0.39	0.26–0.58	Gamma	Reck et al. [12]
mTTD in men: RWE, months	12.3	–	Exponential	Yiu et al. [13]
Hazard ratio for disease progression in women: RCT	0.75	0.46–1.21	Gamma	Reck et al. [12]
mTTD in women: RWE, months	10.3	–	Exponential	Yiu et al. [13]
OS in patients aged < 65 years: RWE, months	17.9	–	Exponential	Yiu et al. [13]
OS in patients aged ≥ 65 years: RWE, months	12.4	–	Exponential	Yiu et al. [13]
OS in men: RWE, months	12.1	–	Exponential	Yiu et al. [13]
OS in women: RWE, months	14.8	–	Exponential	Yiu et al. [13]
*Chemotherapy combination treatments, probability*				
Carboplatin plus pemetrexed	0.444	–	Dirichlet	Reck et al. [10]
Cisplatin plus pemetrexed	0.248	–	Dirichlet	Reck et al. [10]
Carboplatin plus gemcitabine	0.132	–	Dirichlet	Reck et al. [10]
Cisplatin plus gemcitabine	0.073	–	Dirichlet	Reck et al. [10]
Carboplatin plus paclitaxel	0.113	–	Dirichlet	Reck et al. [10]
*Severe TRAEs with first line chemotherapy, probability*				
Anaemia	0.193	–	Beta	Reck et al. [10]
Fatigue	0.033	–	Beta	Reck et al. [10]
Neutropenia	0.133	–	Beta	Reck et al. [10]
Thrombocytopenia	0.053	–	Beta	Reck et al. [10]
*Severe TRAEs with first line pembrolizumab monotherapy, probability*				
anaemia	0.013	–	Beta	Reck et al. [10]
Fatigue	0.019	–	Beta	Reck et al. [10]
Skin toxicity	0.039	–	Beta	Reck et al. [10]
*Costs per month, AU$*				
Pembrolizumab (200 mg, every 3 weeks)	10,318	–	Gamma	PBS item 11494Y [27]
Pemetrexed (500 mg, every 3 Weeks)	105	–	Gamma	PBS item 4600D [52]
Folic acid (500mcg per day)	3	–	Gamma	PBS item 10577Q [53]
Hydroxocobalamin (1 mg, every 3 weeks)	8	–	Gamma	PBS item 10584C [54]
Dexamethasone (4 mg, twice daily for 2 days, every 3 weeks)	3	–	Gamma	PBS item 2507Y [55]
Carboplatin (900 mg, every 3 weeks)	202	–	Gamma	PBS item 4309 T [56]
Cisplatin (75 mg/m^2^, every 3 weeks)	127	–	Gamma	PBS item 4319H [57]
Gemcitabine (3000 mg every 4 weeks)	154	–	Gamma	PBS item 4439P [58]
Paclitaxel (200 mg/m^2^ every 3 weeks)	220	–	Gamma	PBS item 4567 J [59]
Docetaxel (75 mg/m^2^, every 3 weeks)	114	–	Gamma	PBS item 10148D [60]
Nivolumab (480 mg, every 4 weeks)	9,561	–	Gamma	PBS item 11153B [61]
Basic supportive care per month*	2,584	–	Gamma	Goldsbury et al. 2020 [62]
TRAEs*				
Anaemia	6,264	4,559–8,235	Gamma	IHACPA 2023 [34]
Fatigue	12,540	9,127–16,486	Gamma	IHACPA 2023 [34]
Neutropenia	12,016	8,746–15,797	Gamma	IHACPA 2023 [34]
Thrombocytopenia	13,633	9,923–17,923	Gamma	IHACPA 2023 [34]
Skin toxicity	11,490	8,363–15,106	Gamma	IHACPA 2023 [34]
*Utility weights*				
First line treatment in NSCLC	0.71	–	Beta	Brown et al. [36]
Second line treatment in NSCLC	0.67	–	Beta	Brown et al. [36]
Basic supportive care in NSCLC	0.58	–	Beta	Khan et al. [37]
Anaemia	− 0.06	–	Beta	Paracha et al. [40]
Fatigue	− 0.07	0.018	Beta	Paracha et al. [40], Nafees et al. [39]
Neutropenia	− 0.09	0.015	Beta	Paracha et al. [40], Nafees et al. [39]
Thrombocytopenia	− 0.06	–	Beta	Paracha et al. [40], Handorf et al. [38]
Skin toxicity	-0.06	–	Beta	Paracha et al. [40], Tabberer et al. [41]

AEs: adverse events; mTTD: median Time to Treatment Discontinuation, used as proxy for PFS [29]; NSCLC: non-small cell lung cancer; OS: overall survival; PFS: progression-free survival; RCT: randomized clinical trial; RWE: real world evidence; SD: standard deviation; TRAEs: treatment related adverse events; *converted to 2024 AU$ using the CCEMG-EPPI Centre cost convertor [35]

The model incorporated grade ≥ 3 treatment-related adverse events (TRAEs) to reflect regimen-specific safety profiles. Probabilities of key TRAEs were obtained from KEYNOTE-024 [10] and applied to the RCT-based pembrolizumab strategy. In the absence of comparative real-world toxicity data, the same TRAE probabilities were applied to the RWE-based pembrolizumab arm.

### Costs

Drug dosing assumptions and direct medical costs associated with drug acquisition, administration, premedication, comedication, and management of key severe TRAEs are summarised in Table 1. Drug-related costs were obtained from the Australian Pharmaceutical Benefits Scheme [33]. Costs associated with the management of severe TRAEs were obtained from the Australian Independent Health and Aged Care Pricing Authority (IHACPA) [34]. All costs were reported in 2024 Australian dollars (AU$ > > US$0.69). Where necessary, historical cost estimates were inflated to 2024 values using the Campbell and Cochrane Economics Methods Group (CCEMG) and the Evidence for Policy and Practice Information and Coordinating Centre (EPPI-Centre) cost converter [35]. Currency conversions are reported for contextual reference only.

### Utility weights

Health outcomes were measured in quality-adjusted life years (QALYs). Health-state utility values reflecting health-related quality of life (HRQoL) were obtained from published literature (Table 1). Utilities were derived from patient-reported outcome measures assessing domains including mobility, self-care, usual activities, pain/discomfort, and anxiety/depression. Utility values were applied by treatment line and assumed to be 0.71 for first-line therapy [36], 0.67 for second-line therapy [36], and 0.58 for basic supportive care [37]. Disutility decrements were applied to account for key severe TRAEs, including anaemia (− 0.06), fatigue, (− 0.07), neutropenia (− 0.09), thrombocytopenia (− 0.06) and skin toxicity (− 0.06) [38–41].

### Analysis

The cost-utility analysis was performed from an Australian health payer perspective using a multi-health-state semi-Markov model implemented in TreeAge Pro HealthCare (TreeAge Software, Williamstown, MA) [42]. Monthly cycles were used to estimate costs (in 2024 AU$) and outcomes expressed in QALYs over a 3-year time horizon, selected to approximate twice the median survival for patients initiating first-line therapy for metastatic NSCLC [43, 44]. Incremental cost-effectiveness ratios (ICERs) were calculated as the difference in discounted costs divided by the difference in discounted QALYs for pembrolizumab monotherapy (RCT- or RWE-based inputs) relative to standard chemotherapy. Costs and health outcomes were discounted at 5% per annum, consistent with the Pharmaceutical Benefits Advisory Committee (PBAC) guidelines for economic evaluations in Australia [45].

Deterministic one-way sensitivity analyses were conducted to examine the influence of key model parameters on results. Overall decision uncertainty was further characterised using probabilistic sensitivity analysis (PSA), based on 10,000 iterations sampling from appropriate parameter distributions. For parameters without published variance estimates, a ± 10–25% range around base-case values was assumed. Additionally, subgroup analysis by age and sex were performed to assess potential heterogeneity in cost-effectiveness. Cost-effectiveness was evaluated against a willingness-to-pay (WTP) threshold of AU$75,000 per QALY, reflecting the upper bound commonly applied by PBAC for new medicines [46].

### Ethics approval

This article is a hypothetical model-based analysis using previously published and/or publicly available data and did not require ethical approval or institutional review board oversight. The model parameters (listed in Table 1) were obtained from publicly accessible sources, and the original studies had obtained the necessary informed consents and ethical approvals.

## Results

### Base case analysis

Table 2 presents the base-case cost-utility results for metastatic non-oncogene addicted NSCLC with PD-L1 TPS ≥ 50%. Compared with platinum-based chemotherapy, pembrolizumab monotherapy was associated with incremental costs of AU$87,399 when informed by RCT data and AU$75,935 when informed by RWE. Incremental QALY gains were 0.23 QALYs in the RCT-based analysis and 0.11 QALYs in the RWE-based analysis. These differences resulted in ICERs of AU$385,561 per QALY gained in the RCT-based scenario and AU$705,729 per QALY gained in the RWE-based scenario.

**Table 2:** Results of the cost-effectiveness analysis

Strategy, base-case analysis	Costs (discounted)	QALYs (discounted)	Incremental costs	Incremental QALYs	Incremental cost-effectiveness ratio	Optimal strategy in PSA* (%)
Chemotherapy: RCT	AU$43,737	0.610	Comparator	Comparator	Comparator	100
Pembrolizumab monotherapy: RCT	AU$131,136	0.836	AU$87,399	0.227	AU$385,561/QALY	0
Pembrolizumab monotherapy: RWE	AU$119,672	0.717	AU$75,935	0.108	AU$705,729/QALY	0

PSA: probabilistic sensitivity analysis; QALYs: quality-adjusted life years; RCT: randomized control trial; RWE: real-world evidence; Rx: treatment; *WTP < AU$75,000/QALY

### Probabilistic sensitivity analysis

**Fig. 2:**
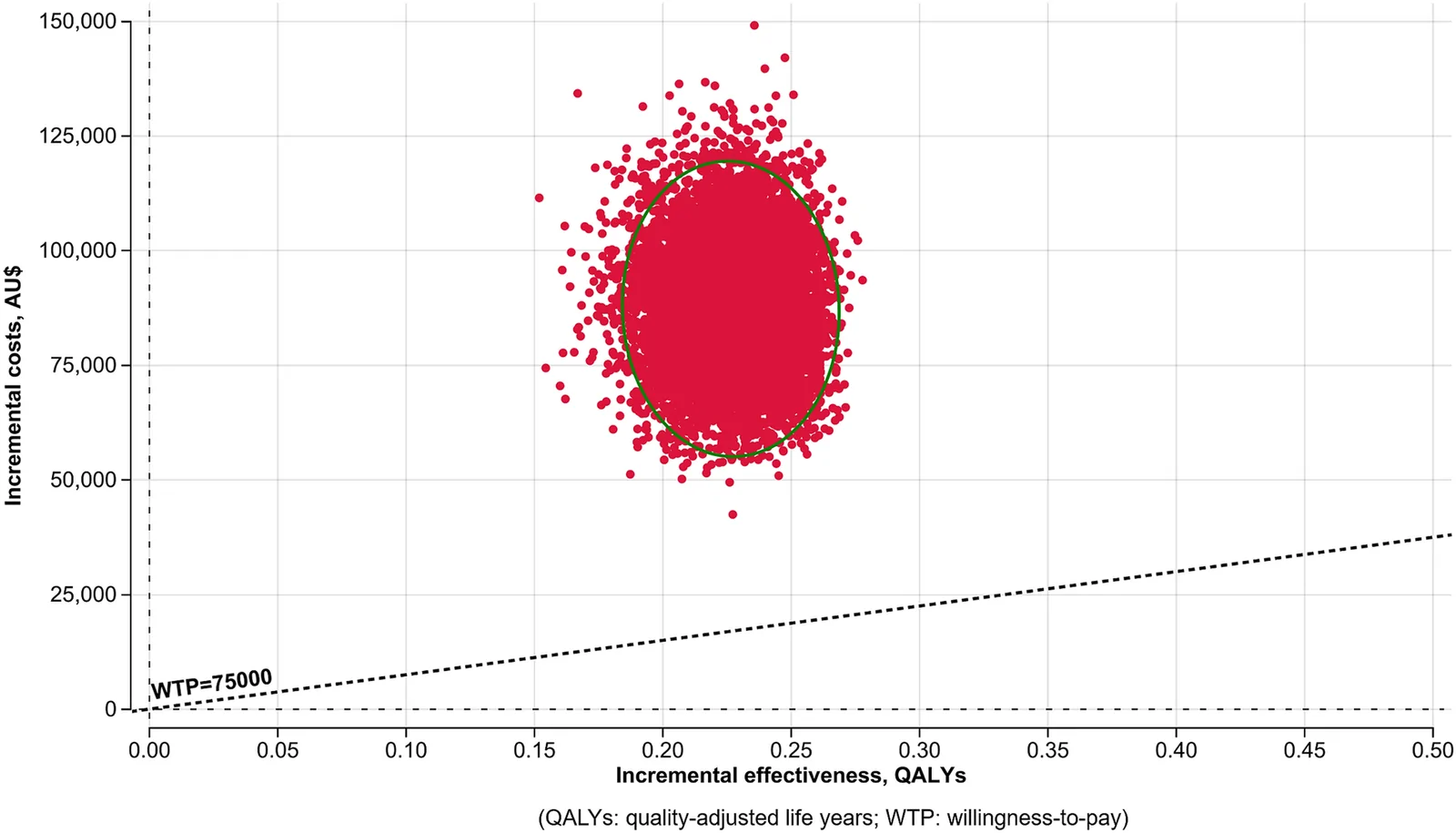
Scatterplot of incremental cost and effectiveness of pembrolizumab monotherapy (RCT) vs. chemotherapy

**Fig. 3:**
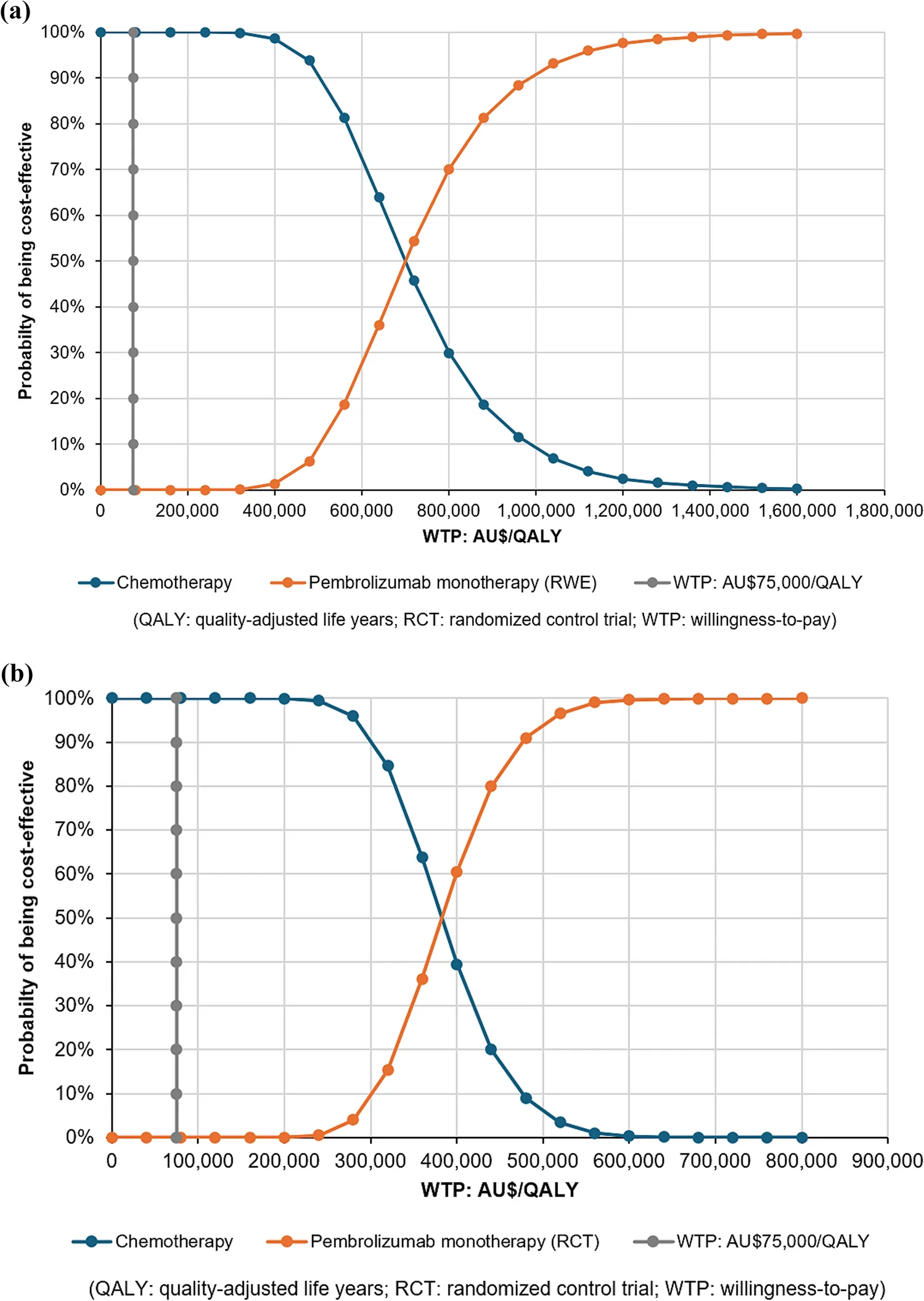
**a** Cost-effectiveness acceptability curves for pembrolizumab monotherapy (RCT) vs. chemotherapy. **b** Cost-effectiveness acceptability curves for pembrolizumab monotherapy (RWE) vs. chemotherapy.

The PSA (10,000 iterations) indicated that pembrolizumab monotherapy had a < 1% probability of being cost-effective at a WTP threshold of AU$75,000 per QALY in both RCT- and RWE-based scenarios. The cost-effectiveness scatterplot derived from the PSA is presented in Fig. 2. Cost-effectiveness acceptability curves (Figs. 3a and b) indicated that WTP thresholds of approximately AU$600,000 per QALY (RCT-based) and AU$1.5 million per QALY (RWE-based) would be required for pembrolizumab monotherapy to achieve ≥ 99% probability of cost-effectiveness.

### Deterministic sensitivity analysis

**Fig. 4:**
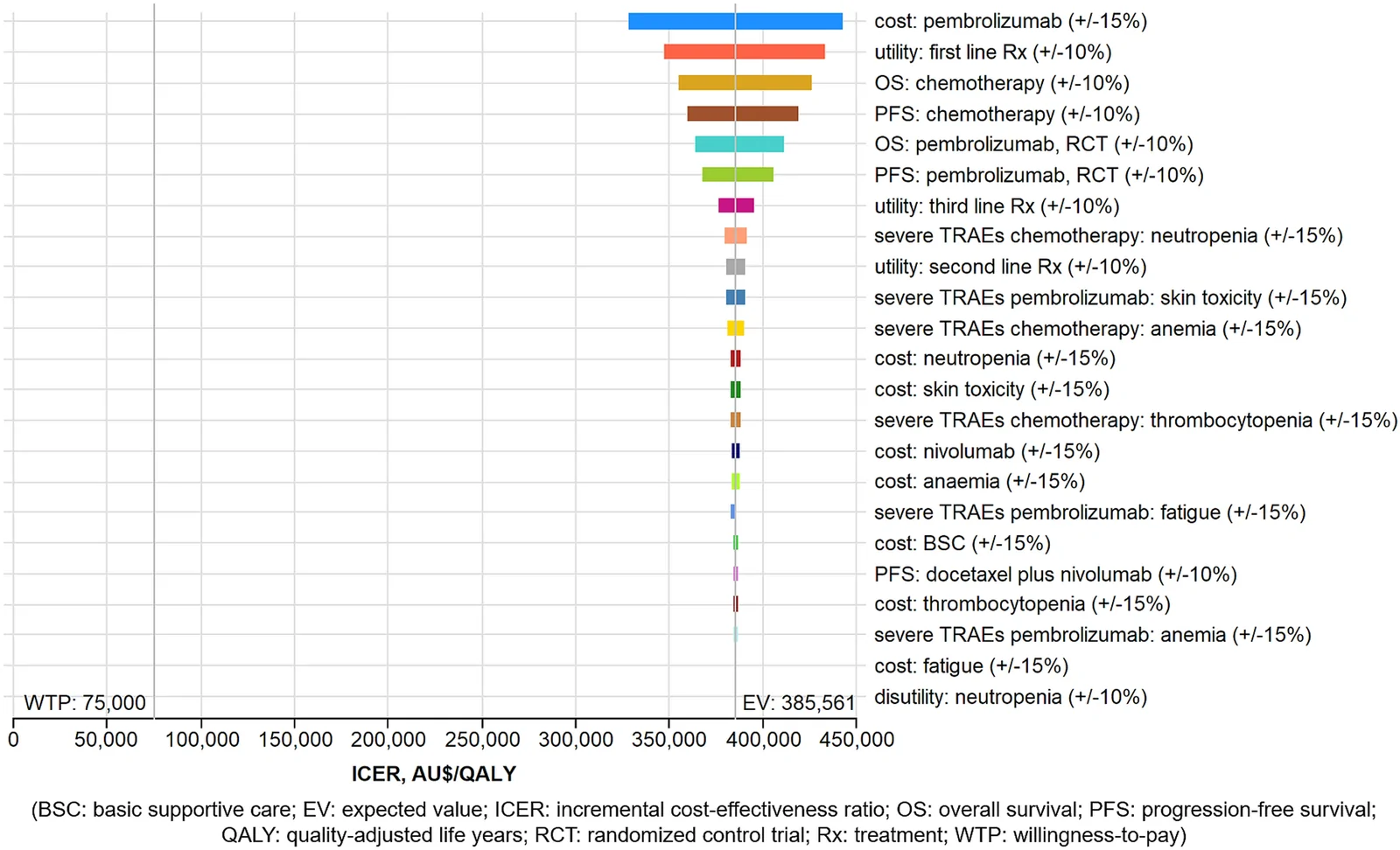
One-way sensitivity analyses of the predictors of the cost-effectiveness of pembrolizumab monotherapy (RCT) vs. chemotherapy

One-way sensitivity analyses identified the cost of pembrolizumab as the most influential parameter affecting the ICER in the RCT-based model (Fig. 4). Other influential parameters included: utility associated with first-line treatment, OS with first-line chemotherapy, PFS with first-line chemotherapy, and OS with first-line pembrolizumab monotherapy. Despite variation across plausible parameter ranges, ICERs in the one-way sensitivity analyses remained substantially above the AU$75,000 per QALY threshold.

### Scenario and subgroup analyses

When a 5-year time horizon was used, there was a marginal 3% drop in the ICER for the RCT-based scenario (AU$374,201/QALY) and 6% drop in the ICER for the RWE-based scenario (AU$667,001/QALY). Additional subgroup results by age and sex are presented in Table S1. The RCT-data showed lower ICERs among patients ≥ 65 years (AU$335,079 per QALY) and among men (AU$307,976 per QALY), compared with the overall cohort. In contrast, RWE-based analyses showed lower ICERs in patients aged < 65 years (AU$438,872 per QALY) and in women (AU$596,957 per QALY). Across all subgroups, ICERs remained above conventional Australian WTP thresholds.

## Discussion

This economic evaluation examined the cost-utility of first-line pembrolizumab monotherapy compared with platinum-based chemotherapy in non-oncogene-addicted metastatic NSCLC with PD-L1 TPS ≥ 50% from an Australian health-payer perspective. The analysis used trial-KEYNOTE-024 outcomes for chemotherapy, with pembrolizumab modelled using either trial-based inputs (RCT scenario) or real-world effectiveness data (RWE scenario). At the upper bound of the WTP threshold commonly applied by the PBAC (AU$75,000 per QALY) [46], pembrolizumab monotherapy was unlikely to be cost-effective, with ICERs of AU$385,561 per QALY in the RCT-based model and AU$705,729 per QALY in the RWE-based model. Probabilistic sensitivity analysis demonstrated a < 1% probability of cost-effectiveness at this threshold. The higher ICER observed in the RWE-based scenario suggests that trial-derived effectiveness estimates may not fully capture outcomes in broader populations treated in routine clinical practice [47].

These findings should not be interpreted as challenging the established clinical efficacy of pembrolizumab in appropriately selected patients. Rather, they highlight the economic implications when survival gains observed under controlled trial conditions are not fully reproduced in real-world settings. The difference in median OS between the trial-based and real-world analyses (26.3 vs. 13.2 months) appears to largely account for the divergence in ICER estimates. The apparent differences in PFS between the trial-based and real-world scenarios should be interpreted cautiously, as the real-world estimates are derived from time-to-treatment discontinuation and are not directly comparable to radiologically assessed PFS from KEYNOTE-024. The primary driver of the observed ICER differences was reduced OS in the RWE scenario, which resulted in substantially lower QALY gains despite concurrent reductions in treatment costs. Shorter OS in routine practice may reflect differences in patient selection, clinical characteristics, and treatment pathways compared with the highly selected KEYNOTE-024 population [10]. Such discrepancies between trial efficacy and real-world effectiveness are increasingly recognized in oncology and are particularly relevant when long-term survival drives economic value [47, 48]. These findings should therefore be interpreted in the context of a scenario analysis in which real-world effectiveness estimates for pembrolizumab are substituted for trial-based inputs, recognizing that this does not represent a fully real-world comparative cost-effectiveness evaluation.

The observed differences in outcomes between the KEYNOTE-024 trial population and the Australian real-world cohort are consistent with known variations in patient characteristics between clinical trials and routine practice. Compared with KEYNOTE-024, the real-world cohort was older (median age 74 vs. 64.5 years) and had a higher comorbidity burden as measured by the Rx-Risk score (median 5.0, indicating moderate comorbidity). In addition, a substantial proportion of patients had steroid-responsive conditions and prior corticosteroid exposure, which was not permitted in the KEYNOTE-024 trial. Prior corticosteroid use has previously been associated with reduced survival in this cohort, suggesting potential attenuation of immunotherapy effectiveness in routine practice. These differences may partly explain the observed reduction in overall survival in the real-world analysis and provide clinical context for the divergence in cost-effectiveness results between trial-based and real-world scenarios.

Several cost-effectiveness studies have previously evaluated pembrolizumab monotherapy using data from the KEYNOTE-024 trial. The results of the current study’s RCT-based analysis were broadly consistent with studies conducted in the United Kingdom [20], China [21], and Singapore [22] which concluded that pembrolizumab monotherapy was not cost-effective at locally applied WTP thresholds. In contrast, analyses from the United States [14], Switzerland [17], France [18], and Hong Kong [19] reported more favourable cost-effectiveness conclusions. Variability across studies likely reflects differences in healthcare system costs, drug pricing arrangements, modelling approaches, time horizons, and WTP thresholds. For example, although similar ICER magnitudes were reported in analyses from the United States [14] and China [21], pembrolizumab was considered cost-effective in the United States but not in China due to substantial differences in national WTP thresholds (US$150,000/QALY vs. US$26,481/QALY, respectively). These observations underscore the importance of interpreting cost-effectiveness results within their specific policy and healthcare system contexts.

From a methodological perspective, many prior evaluations employed partitioned survival models, which, although widely accepted in oncology economic analyses, rely on structural assumptions that may affect long-term projections of survival and QALYs [49]. In contrast, the semi-Markov state-transition framework applied in the present study enabled explicit modelling of post-progression treatment pathways and health-state transitions. Differences in model structure, extrapolation methods, and health-state utility assumptions may partly account for heterogeneity in ICER estimates reported across studies.

Compared with the numerous trial-based economic evaluations, few studies have incorporated RWE into cost-effectiveness modelling for immune checkpoint inhibitors in advanced NSCLC. In a previous study [50], we examined the cost-effectiveness of first-line pembrolizumab plus platinum-based chemotherapy compared with chemotherapy alone in an unselected mNSCLC population irrespective of PD-L1 status. That analysis incorporated efficacy data from the KEYNOTE-189 trial and RWE from a population-based study by Yiu et al. [51], covering patients across all PD-L1 subgroups (< 1%, 1–49% and ≥ 50%). In contrast, the present study focused on pembrolizumab monotherapy patients with PD-L1 TPS ≥ 50%, a clinically distinct subgroup for whom monotherapy is the recommended first-line treatment [5, 8, 9]. Accordingly, the two analyses address different clinical and reimbursement questions and should be considered complementary. The pharmacoeconomic evaluation supporting reimbursement of pembrolizumab monotherapy for NSCLC in Australia relied primarily on KEYNOTE-024 data and reported an ICER within the PBAC’s implicit WTP threshold of AUD$45,000-$75,000 per QALY [46]. In contrast, our analysis demonstrates that applying real-world survival outcomes results in less favourable cost-effectiveness estimates. With drug prices and utility weights held constant, and similar downstream disease PFS assumptions across scenarios, the marked difference in OS (26.3 months vs. 13.2 months) largely accounts for the higher ICER in the RWE scenario. Although incremental costs were lower in the RWE-based model due to shorter treatment duration and earlier mortality (AU$75,935 vs. AU$87,399), incremental QALY gains were substantially reduced (0.11 vs. 0.23), resulting in a higher ICER. These findings illustrate how differences in survival outcomes can materially influence economic conclusions and emphasize the importance of incorporating RWE alongside RCT evidence when evaluating long-term value.

Although conducted within the Australian healthcare system, these observations have broader implications for health technology assessment internationally. Differences in willingness-to-pay thresholds, drug pricing arrangements, and reimbursement frameworks across jurisdictions mean that absolute cost-effectiveness results are context-specific and not directly transferable between countries. However, the observed divergence between trial-based and real-world effectiveness scenarios is likely to be relevant across healthcare systems where reimbursement decisions are sensitive to survival outcomes and treatment duration. From a policy perspective, including in the Australian PBAC context, these findings highlight the importance of addressing uncertainty in real-world effectiveness at the time of listing, with potential implications for pricing negotiations, managed entry agreements, and post-listing evidence generation to support reassessment of long-term value over time.

### Strengths and limitations

To our knowledge, this is among the first studies to directly compare RCT- and RWE-informed cost-effectiveness estimates for pembrolizumab monotherapy in advanced NSCLC. The analysis integrated five-year follow-up data from KEYNOTE-024 [10] with contemporary nationwide Australian RWE [13], providing a comprehensive assessment of economic value under both controlled trial conditions and routine clinical practice. Deterministic and probabilistic sensitivity analyses characterized both parameter and decision uncertainty, enhancing the robustness and policy relevance of the findings.

Several limitations should be considered when interpreting these findings. First, certain model inputs, including health-state utilities and post–second-line progression parameters, were derived from published literature rather than directly from the trial or real-world datasets. Further, while PFS was used to assess disease progression in RCT-based data, the RWE-data relied on mTTD as a pragmatic proxy for disease progression [29]. Although sensitivity analyses suggested limited influence of our assumptions on overall conclusions, some uncertainty remains. Second, the RWE scenario applies real-world effectiveness estimates to the pembrolizumab arm while retaining KEYNOTE-024 trial-based chemotherapy outcomes due to the absence of a contemporaneous real-world chemotherapy cohort, resulting in a hybrid comparative framework. This preserves consistency with the comparator used in the pivotal KEYNOTE-024 trial that informed reimbursement decisions. Accordingly, this scenario should be interpreted as a scenario analysis evaluating the impact of substituting trial-based effectiveness with real-world estimates for pembrolizumab, rather than a fully real-world comparative effectiveness assessment. Third, real-world survival estimates may be subject to unmeasured confounding, including differences in patient characteristics and access to subsequent therapies relative to the randomised trial population. The real-world cohort used to derive pembrolizumab effectiveness estimates did not contain individual-level PD-L1 expression data and therefore reflects a heterogeneous mNSCLC population rather than a strictly PD-L1 TPS ≥ 50% subgroup. Although PD-L1 TPS was not available in administrative claims data, pembrolizumab monotherapy is predominantly prescribed in PD-L1-high disease in the Australian PBS setting, suggesting that the real-world cohort is likely enriched for this subgroup. This introduces uncertainty in mapping real-world estimates to the trial-defined PD-L1 ≥ 50% population. Immune-related adverse events may be under-captured in claims data due to lack of inpatient medication recording; therefore, trial-based adverse event estimates were used in the model. Scenario and sensitivity analyses suggest this uncertainty does not materially alter conclusions. Fourth, cost input was based on publicly available sources. IHACPA reports bundled hospitalisation costs for grouped adverse event categories (e.g., neutropenia within broader haematological complications), which may limit condition-specific precision. In addition, PBS list prices were applied, as confidential rebates are not publicly disclosed. As a result, estimated ICERs may differ from confidential values used in jurisdictional reimbursement assessments. Finally, extending the model horizon beyond three years is unlikely to materially alter conclusions, as > 99% of patients in both the pembrolizumab and chemotherapy strategies were projected to have died by this time point. Scenario analysis using a 5-year time horizon demonstrated only modest changes in cost-effectiveness estimates, suggesting limited sensitivity of results to further time horizon extension. Nevertheless, long-term extrapolation of survival curves remains inherently uncertain, particularly for immunotherapies where a small proportion of patients may achieve durable survival.

## Conclusion

In this Australian health-payer analysis, first-line pembrolizumab monotherapy for advanced non-oncogene-addicted NSCLC with PD-L1 TPS ≥ 50% was unlikely to be cost-effective under current pricing and WTP thresholds. Cost-effectiveness estimates were less favourable when informed by real-world survival outcomes compared with randomized trial data. These findings suggest that cost-effectiveness estimates may vary depending on whether trial-based or real-world effectiveness inputs are applied within the modelling framework.

## Supplementary Information

Below is the link to the electronic supplementary material.

Supplementary file1 (DOCX 26 KB)

## Data Availability

No datasets were generated or analysed during the current study.
