# Supplementary material for: Cost-effectiveness of first-line pembrolizumab monotherapy in PD-L1–high metastatic non-small cell lung cancer in Australia: evidence from trials and real-world practice
Source: Int J Clin Pharm. 2026 Jul 27;48(6):2237–49. doi: 10.1007/s11096-026-02202-3 (PMC13731505; doi:10.1007/s11096-026-02202-3)
Supplement: Supplementary file 1 [file 11096_2026_2202_MOESM1_ESM.docx]

# Table S1. Results of the cost-effectiveness analysis by subgroups

| Strategy, base-case analysis | Costs (discounted) | QALYs (discounted) | Incremental costs | Incremental QALYs | Incremental cost-effectiveness ratio | Optimal strategy in PSA* |
| --- | --- | --- | --- | --- | --- | --- |
| Age <65 years |  |  |  |  |  |  |
| Chemotherapy: RCT^†^ | AU$43,737 | 0.610 | comparator | comparator | comparator | 100% |
| Pembrolizumab monotherapy: RCT | AU$127,614 | 0.789 | AU$83,877 | 0.180 | AU$466,655/QALY | 0% |
| Pembrolizumab monotherapy: RWE | AU$135,029 | 0.818 | AU$91,292 | 0.208 | AU$438,872/QALY | 0% |
| Age ≥65 years |  |  |  |  |  |  |
| Chemotherapy: RCT^†^ | AU$43,737 | 0.610 | comparator | comparator | comparator | 100% |
| Pembrolizumab monotherapy: RCT | AU$157,695 | 0.950 | AU$113,958 | 0.340 | AU$335,079/QALY | 0% |
| Pembrolizumab monotherapy: RWE | AU$116,491 | 0.696 | AU$72,754 | 0.087 | AU$840,538/QALY | 0% |
| Men |  |  |  |  |  |  |
| Chemotherapy: RCT^†^ | AU$43,737 | 0.610 | comparator | comparator | comparator | 100% |
| Pembrolizumab monotherapy: RCT | AU$172,987 | 1.029 | AU$129,250 | 0.420 | AU$307,976/QALY | 0% |
| Pembrolizumab monotherapy: RWE | AU$117,279 | 0.696 | AU$73,542 | 0.086 | AU$850,604/QALY | 0% |
| Women |  |  |  |  |  |  |
| Chemotherapy: RCT^†^ | AU$43,737 | 0.610 | comparator | comparator | comparator | 100% |
| Pembrolizumab monotherapy: RCT | AU$109,452 | 0.689 | AU$65,715 | 0.079 | AU$827,238/QALY | 0% |
| Pembrolizumab monotherapy: RWE | AU$121,906 | 0.740 | AU$78,169 | 0.131 | AU$596,957/QALY | 0% |

PSA: probabilistic sensitivity analysis; QALYs: quality-adjusted life years; RCT: randomized control trial; RWE: real-world evidence; Rx: treatment; *WTP <AU$75,000/QALY; **^†^**whole cohort-based estimation
